# DEVELOPMENT AND PRODUCTION OF AN ORIGINAL MUSICAL WITHIN A RETIREMENT COMMUNITY

**DOI:** 10.1093/geroni/igad104.0910

**Published:** 2023-12-21

**Authors:** Philip Sloane

**Affiliations:** University of North Carolina, Chapel Hill, North Carolina, United States

## Abstract

In 2021-23 residents of a continuing care retirement community (CCRC) conceptualized, wrote, produced, and presented an original musical about life in a senior community. This presentation will describe that process, illustrating it with a video presentation by the 83-year-old CCRC resident who organized and coordinated the process, video clips of rehearsals and of interviews with the songwriters and performers, and video performances of four songs from the musical. Among the gerontological issues to be highlighted include: deciding which song topics the community would deem acceptable and unacceptable to address in the production; accommodating the physiological limits of the cast (e.g., reduced vocal ranges with age) and audience (e.g., hearing impairment and limited bladder capacity); and adjusting to absences due to illnesses, medical appointments, family events, and travel among the organizers and performers. A portion of this presentation will center around four original songs written and performed by seniors as part of the musical: “Rules of the Road” (about the informal “do’s and don’ts” of living in a retirement community); “The Ladies of Lavender Lane” (about groups of widows who have banded together for support); “The Rhythm of our Lives” (about how the community deals with the high frequency of resident deaths); and “I’m Just a Little Worried about Eddie” (about early dementia). The development of each song will be summarized, including the process by which the song went from idea to production, and a video of the song during a performance.

